# A male patient with proptosis and palatal perforation: A diagnostic challenge

**DOI:** 10.1016/j.radcr.2025.04.135

**Published:** 2025-05-28

**Authors:** Peyman Mottaghi, Sam Mirfendereski, Farshad Riahi, Shahin Rajaeih

**Affiliations:** a Department of Rheumatology, Isfahan University of Medical Sciences, Isfahan, Iran; b Department of Radiology, Isfahan University of Medical Sciences, Isfahan, Iran; c ENT and Head and Neck Research Center and Department, The Five Senses Health Institute, Firoozgar Hospital, Iran University of medical Sciences, Tehran, Iran

**Keywords:** Limited Wegener's granulomatosis, Palatal perforation, Proptosis, Vasculitis

## Abstract

Granulomatosis with polyangiitis (Wegener's granulomatosis), affects many systems and can show up in a variety of ways. Sometimes, only a few organs are affected. Rare complications of this vasculitis syndrome include a retroorbital pseudotumor with proptosis due to chronic tissue inflammation, as well as necrosis with subsequent palate perforation. We present a 59-year-old male who had multiple ophthalmologic evaluations for chronic pain, redness, and proptosis in the right eye. During the workup for eye problems, he underwent surgery for chronic right nasal obstruction and bloody discharge. After 2 weeks, he noticed pain and ulceration in his hard palate that rapidly transformed into a perforation of the palate and was finally referred to a rheumatologist due to increased erythrocyte sedimentation rate. A CT scan was requested for the patient, which showed an orbital mass and palatal perforation. A biopsy of the mass behind the eye showed that it was inflamed across the membrane and had fibrinoid necrosis, which is a sign of necrotizing vasculitis.

## Introduction

Wegener's granulomatosis (WG) is a systemic inflammatory disease characterized by necrosis, granuloma formation, and vasculitis. It affects the upper and lower respiratory tracts and kidneys, with ANCA present in 80-90% of patients. WG can be controlled with glucocorticoid and immunosuppressive therapies [1]. Ophthalmologic involvement is a significant cause of morbidity, occurring in 50% of WG patients [2]. Oral features include delayed healing of extraction wounds, lingual necrosis, osteonecrosis of the palate, oral-antral fistulae, swelling and desquamation of the lips and salivary gland enlargement [3].

Both palatal perforation and proptosis are diagnostic challenges for ophthalmologists and otolaryngologists. Both entities rarely occur in the same patient, and the physician should be aware of systemic disease in such a case. We present a male patient with the development of these complaints for whom the same etiopathogenesis for both conditions was overlooked.

## Case presentation

A 58-year-old male was referred to a rheumatologist due to an elevated erythrocyte sedimentation rate (ESR) and a nonhealing palatal perforation. His medical history included severe rhinosinusitis and a surgical intervention for septal deviation 2 months prior to admission. The surgery was performed to alleviate chronic nasal obstruction and bloody discharge, which had persisted for an extended period.

The initial presentation of the illness involved a gradual onset of pain in the hard palate area, which began a few weeks postsurgery. This pain was accompanied by erythema and ulceration, leading to a significant disruption in his quality of life. Concurrently, the patient had a history of multiple ophthalmologic visits for persistent pain and erythema in the right eye, which had not resolved with initial treatment. However, the ophthalmological examination revealed a 20/20 visual acuity in the right eye. Further examination using biomicroscopy showed normal pupillary responses, a healthy front portion of the right eye, normal intraocular pressure, and an eye fundus that was within typical parameters.

We reviewed the patient's family history for autoimmune diseases but found no specific case.

**Fig. 1:**
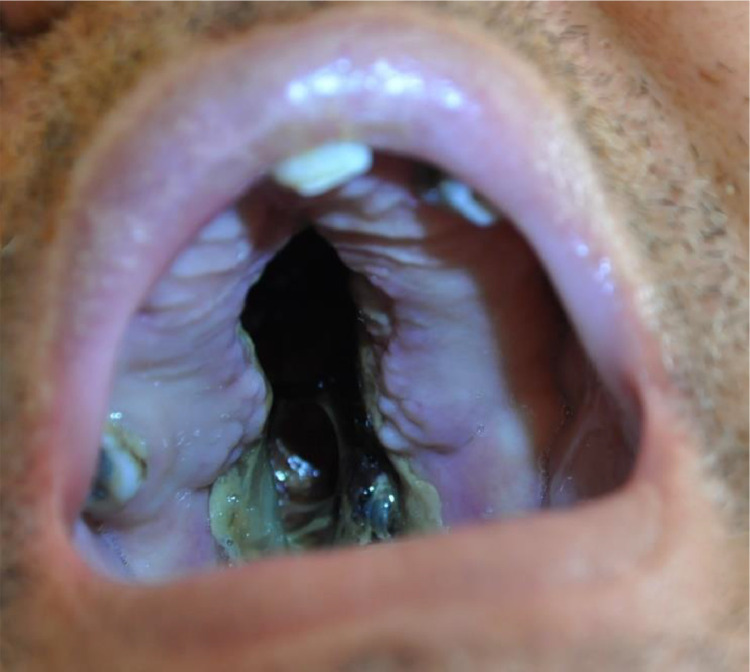
Hard palate perforation by limited Wegener’s granulomatosis.

On examination, the oral cavity revealed a large perforation of the hard palate (Fig. 1). The right nasal passage was obstructed, causing significant difficulty in nasal breathing. Clinical findings included proptosis of the right eye and signs of dacryocystitis.

The blood tests showed that the white blood cell count was 9200/mm³, with 70.5% neutrophils, 2.5% eosinophils, and 27.0% lymphocytes; the hemoglobin level was 13.7 g/dL; the platelet level was 480,000/mm³; the C-reactive protein (CRP) level was 12.2 mg/dL (normal is less than 0.6 mg/dL); and the ESR was 72 mm/hr. Other laboratory tests, including urinalysis, serum creatinine, antinuclear antibodies (ANA), anti-neutrophil cytoplasmic antibodies (ANCA), and angiotensin-converting enzyme (ACE) levels, were within normal limits (Table 1).

**Table 1:** Laboratory findings of our case.

Test	Result	Normal ranges
BS (mg/dL)	95	70-130
BUN (mg/dL)	19	6-25
Cr (mg/dL)	0.9	0.6-1.3
Na (mEq/L)	138	136-145
K (mEq/L)	4.2	3.8-5
RBC ( × 10^3^/µL)	4.13	4.1-5.1
WBC (/µL)	9200	4000-11,000
Hb (g/dL)	13.7	12.3-15.3
Hct (%)	37.4	35.9-44.6
Neut (%)	70.5	40%-60%
Lymph (%)	27	20%-40%
Eosin (%)	2.5	1–4%
Plat (/mm^3^)	480,000	150,000-400,000
ESR (mm/hr)	72	0-20
CRP(mg/dL)	12.2	<0.6
TSH (mlu/L)	0.86	0.3-5.0
Bili T (mg/dL)	0.9	0.1-1.2
Bili D (mg/dL)	0.2	0.1- 0.2
AST (IU/L)	22	<31.0
ALT (IU/L)	26	<31
ALK (IU/L)	159	70-260
ANA (U)	0.34	≤1.0 U
Anti-dsDNA (IF)	<1:10	Neg: < 1:10 Pos: > 1:10
P–ANCA (IU/ml)	4	<5
C–ANCA (IU/ml)	<3	<3
ACE (IU/L)	10.4	8-22

Blood sugar (mg/dL); Blood urea nitrogen (mg/dL); Creatinine (mg/dL); Sodium (mEq/l); Potassium (mEq/l); Red blood cells (Mil/mm³); White blood cells (/m³); Hemoglobin (g/dL); Hematocrit (%); Neutrophils (%); Lymphocytes (%); Eosinophils (%); Platelets (/mm³); Erythrocyte sedimentation rate (mm/hr); C-reactive protein (mg/dL); Thyroid stimulating hormone (mIU/L); Total bilirubin (mg/dL); Direct bilirubin (mg/dL); Aspartate aminotransferase (IU/L); Alanine aminotransferase (IU/L); Alkaline phosphatase (IU/L); Antinuclear antibody (U); Antidouble stranded DNA (IF); Perinuclear antineutrophil cytoplasmic antibodies (IU/ml); Cytoplasmic antineutrophil cytoplasmic antibodies (IU/ml); Angiotensin-converting enzyme (IU/L).

**Fig. 2:**
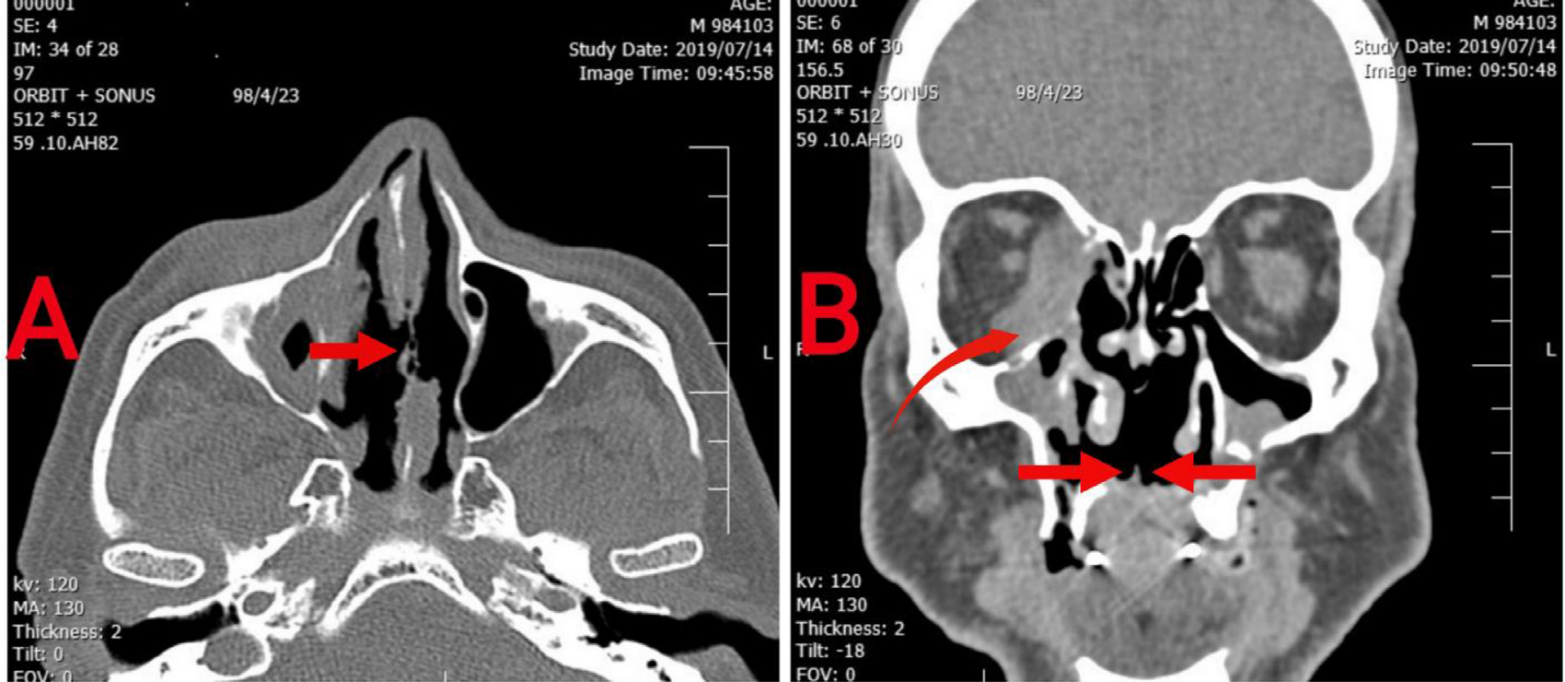
(A) axial image of nasal cavity shows severe erosion of nasal septum (red arrow); (B) coronal image shows absence of hard palate which allows communication between oral and nasal cavity (red arrows). Also mass like lesion in medial side of right orbit (curved arrow) and bilateral maxillary sinusitis is noted.

Imaging studies included a chest CT scan, which showed no abnormalities. However, a CT scan of the paranasal sinus and orbit revealed significant damage to the nasal septum, a soft tissue mass in the right orbit, and a palatal perforation (Fig. 2). A biopsy of the orbital mass indicated transmural inflammation and fibrinoid necrosis, consistent with necrotizing vasculitis.

The management approach involved a multidisciplinary team, including rheumatology and otolaryngology specialists. The patient was placed on pharmacological treatment under the care of a rheumatologist. Also, to close the palatal defect, prevent regurgitation during feeding, and enhance the speech process, a palatal obturator prosthesis was utilized. He was monitored closely for potential complications and response to treatment.

Over the following weeks, the patient's symptoms gradually improved. The proptosis and nasal obstruction began to resolve. Follow-up evaluations indicated a decrease in inflammatory markers, and the patient reported significant relief from pain.

## Discussion

There are many causes for an abnormal protrusion of the eyeball (proptosis), but the 2 most common and important are malignancy and inflammatory diseases involving the orbit [4]. Problems in the paranasal sinuses and nose can spread to the orbit, and as a result, proptosis may be a sign of the problem in this area. Systemic inflammatory processes, including necrotizing vasculitis, constitute a group of serious causes of proptosis and nasal lesions [4]. Ulceration of the palate and acquired palatal perforation are also rare but serious entities that can be caused by various etiologies, including infectious and systemic inflammatory diseases [5]. The nonspecific nature of the early presentation of proptosis or palatal perforation complicates the diagnosis process. We present a case of proptosis with concomitant palatal perforation caused by limited granulomatosis with polyangiitis.

The limited form of granulomatosis and polyangiitis (GPA), formerly known as Wegener’s granulomatosis, is a necrotizing vasculitis localized to the orbit, sinuses, and upper airway [6]. Vasculitis of granulomatosis and polyangiitis affects many body systems and can show up in a variety of ways. Sometimes, only a few organs are affected. The cytoplasmic antineutrophilic cytoplasmic antibody (c-ANCA) test is useful, but it might not show up in cases of the disease that are limited to one area [7]. It is important to report this case because it has a very unusual set of symptoms: proptosis, palatal perforation, and negative ANCA serology.

Ocular diseases, such as scleritis, uveitis, dacryocystitis, lid edema, diplopia, decreased vision, and proptosis, may be the sole presenting manifestation in 8%-16% of GPA cases [8]. Orbital involvement and eye problems were reported in about 30%-50% of the patients, and 14%-30% have bilateral disease. Proptosis is one of the most frequent manifestations of orbital involvement seen in up to 22% of all cases and is often caused by extension of pathology from adjacent sinuses [8].

Head and neck involvement occurs in about 80% of the patients with GPA, but about 5% of the patients experience only localized involvement in the sinonasal area [6]. Around 2% of GPA cases present with oral lesions, and about 5%-10% of all patients develop these lesions during the clinical course [9]. Palatal mucosal ulceration and perforation is a rare form of oral lesion [6] in GPA, and other causes include cocaine abuse, trauma, lymphoma, and infections such as mucormycosis [5], which were ruled out in our patient.

In our case, a tissue biopsy reveals transmural inflammation with fibrinoid necrosis, but orbital biopsies rarely display the classic features of vasculitis [10]. ANCA tests are often not elevated in limited forms of GPA and may not be useful in these settings [6], as in our case.

## Conclusion

Severe morbidity, including proptosis or palatal perforation, may occur if there is a delay in diagnosis of GPA. The goal of presenting this case is to expose clinicians to the rare manifestations of this vasculitis to aid in early diagnosis and treatment.

## Patient consent

Written informed consent for the publication of this case report was obtained from the patient.
